# Diabetes induces stable intrinsic changes to myeloid cells that contribute to chronic inflammation during wound healing in mice

**DOI:** 10.1242/dmm.012237

**Published:** 2013-09-18

**Authors:** Pauline Bannon, Sally Wood, Terry Restivo, Laura Campbell, Matthew J. Hardman, Kimberly A. Mace

**Affiliations:** 1 The Healing Foundation Centre, University of Manchester, Manchester, M13 9PT, UK; 2 University of Texas, San Antonio Health Science Center, San Antonio, TX 78229, USA

## Abstract

Acute inflammation in response to injury is a tightly regulated process by which subsets of leukocytes are recruited to the injured tissue and undergo behavioural changes that are essential for effective tissue repair and regeneration. The diabetic wound environment is characterised by excessive and prolonged inflammation that is linked to poor progression of healing and, in humans, the development of diabetic foot ulcers. However, the underlying mechanisms contributing to excessive inflammation remain poorly understood. Here we show in a murine model that the diabetic environment induces stable intrinsic changes in haematopoietic cells. These changes lead to a hyper-responsive phenotype to both pro-inflammatory and anti-inflammatory stimuli, producing extreme M1 and M2 polarised cells. During early wound healing, myeloid cells in diabetic mice show hyperpolarisation towards both M1 and M2 phenotypes, whereas, at late stages of healing, when non-diabetic macrophages have transitioned to an M2 phenotype, diabetic wound macrophages continue to display an M1 phenotype. Intriguingly, we show that this population predominantly consists of Gr-1^+^ CD11b^+^ CD14^+^ cells that have been previously reported as ‘inflammatory macrophages’ recruited to injured tissue in the early stages of wound healing. Finally, we show that this phenomenon is directly relevant to human diabetic ulcers, for which M2 polarisation predicts healing outcome. Thus, treatments focused at targeting this inflammatory cell subset could prove beneficial for pathological tissue repair.

## INTRODUCTION

The inflammatory response to injury is an ancient and evolutionarily conserved process that involves recruitment of circulating cells to damaged tissue to neutralise invading pathogens and remove local debris. This process must be tightly regulated in order to prevent an excessive or aberrant response, which could further damage surrounding tissue. Chronic inflammation, a serious condition underlying many disease complications, is the result of a dysregulated and excessive inflammatory response. The contribution of inflammation to healthy wound healing remains somewhat controversial. Some studies have suggested that inflammation might impair the tissue repair process (Cooper et al., 2004; Dovi et al., 2003), whereas more recent conditional macrophage depletion studies have demonstrated their essential and positive contribution to adult cutaneous repair (Lucas et al., 2010; Mirza et al., 2009). However, it remains unclear whether these positive inflammatory cell roles translate into pathological wound healing environments, such as in diabetes. For example, we have recently shown that Gr-1^+^ CD11b^+^ cells isolated from diabetic wounds show aberrant gene expression and behaviour, and fail to promote neovascularisation (Mahdipour et al., 2011). This dysfunctional phenotype might be due to abnormalities in myeloid cell development, differentiation or environmentally induced activation.

Myeloid cells can be differentially activated or polarised by the local environment into different states associated with Th1 and Th2 cytokines. These myeloid cells are then referred to as classically activated (M1) or alternatively activated (M2). These classifications are derived from *in vitro* studies eliciting bone marrow (BM)-derived or peritoneal macrophages into activated states with pro-inflammatory factors, such as interferon gamma and bacterial lipopolysaccharides for classical/M1 activation, or interleukin (IL)-4/IL-13 stimulation plus blockade of interferon gamma for alternative/M2 activation (Gordon, 2003).

*In vivo* analyses of inflammatory cells have suggested that, although this classification scheme might not precisely align *invivo*-derived myeloid cells with their *in vitro* counterparts, it is a useful model for describing myeloid cells obtained from different environments. For example, gene expression profiling reveals that murine wound macrophages show a more mixed polarisation phenotype compared with macrophages assayed *in vitro*, but overall demonstrate an M1-type profile early in healing, switching to an M2-type profile in the later, pro-repair phase of wound healing (Daley et al., 2010). Two recent studies in diabetic rats and mice suggest that inflammatory cell activation is aberrant in the diabetic wound environment, because gene expression analyses of inflammatory cell activation markers show a prolonged M1-type signature (Miao et al., 2012; Mirza and Koh, 2011).

Even less is known about the regulation of M1 and M2 macrophage polarisation and behaviour *in vivo*. An open question is whether M1 and M2 macrophages are pre-determined prior to tissue recruitment, or whether this is solely determined by the local environment. Jenkins et al. demonstrated that, in a lung injury model, proliferation of tissue-resident macrophages gives rise to the majority of M2-type macrophages (Jenkins et al., 2011), suggesting that these cells might not be recruited at all, but develop in response to environmental cues. By contrast, the majority of M2 macrophages in tumours are recruited (Acero et al., 1984; Evans and Cullen, 1984); thus, the mechanisms regulating M2 macrophage development seem to be very much context dependent. Interestingly, despite potential differences in the origin of M2 macrophages, one recent study shows that even under different pathological conditions M2 macrophages share a common gene expression signature (Ghassabeh et al., 2006), suggesting a common underlying M2 phenotype.

**TRANSLATIONAL IMPACT**

**Clinical issue**

Worldwide, over 220 million people have diabetes. The condition often leads to the development of chronic wounds, which can ultimately necessitate limb amputation. It is estimated that in the UK alone there are over 100 amputations per week as a result of chronic wounds in diabetics. Current treatments for diabetes-associated wounds are ineffective owing to a poor understanding of the underlying mechanisms causing chronic wounds, leading to a substantial burden on the healthcare system and high numbers of morbidity and mortality. Chronic inflammation, a result of excessive and inappropriate inflammatory responses, is thought to contribute to the pathological wound-healing environment associated with diabetes. However, the cellular mechanisms involved remain unclear.

**Results**

In this study, the authors used a well-characterised mouse model of type 2 diabetes to determine how the diabetic environment affects the differential activation (or polarisation) of myeloid progenitors. They demonstrate that diabetes induces stable cell-intrinsic changes that pre-prime myeloid cells to hyperpolarise towards ‘M1’ pro-inflammatory macrophages and ‘M2’ anti-inflammatory macrophages during the early stages of wound healing. In line with this, there is initially a hyper-responsive phenotype to both pro-inflammatory and anti-inflammatory stimuli. However, during the late stages of wound healing, extrinsic factors inhibit the development of M2 anti-inflammatory macrophages; thus, the pro-inflammatory response mediated by M1 macrophages is prolonged. By contrast, wounds of non-diabetic mice transitioned to a pro-healing phenotype characterised by more M2 macrophages and fewer M1 macrophages within the same timeframe. The authors demonstrate that failure of M2 macrophage development is predictive of non-healing foot ulcers in humans with diabetes.

**Implications and future directions**

This work provides the first evidence that cell-intrinsic factors in the diabetic environment shape the polarisation of recruited inflammatory cells. Intriguingly, extrinsic factors in the diabetic wound environment then act to prolong the pro-inflammatory response, which could contribute to the lack of healing. This conclusion is supported by the authors’ analysis of human diabetes-associated non-healing. The study demonstrates the potential for intrinsic defects in inflammatory cells versus a deregulated wound environment to influence wound healing. Future studies should focus on specifying the cell-intrinsic factors (such as transcription factor expression or chromatin regulation) that pre-prime myeloid cells to generate a pro-inflammatory response, and on determining which cell types play a role in prolonging the M1 macrophage phenotype in the diabetic wound environment. Based on these findings, it could be possible to develop therapies that re-programme inflammatory cells directly to prevent or reverse chronic wound development, or to promote healing at the later stages.

In the present study, we investigated whether the diabetic environment alters myeloid cell differentiation and/or M1/M2 polarisation potential via cell-intrinsic or -extrinsic factors. Diabetes-induced ‘pre-priming’ of myeloid progenitors towards a pro-inflammatory phenotype with aberrant M1/M2 polarisation kinetics would be of clear therapeutic relevance. Specifically, if myeloid progenitors are pre-determined early to become M1/pro-inflammatory macrophages, then therapies could focus on myeloid cell development, whereas if cell-extrinsic factors are dominant, then therapies aimed at correcting these local signals should be more effective.

## RESULTS

### Diabetic wounds display aberrant markers of M1 and M2 polarisation

**Fig. 1.**
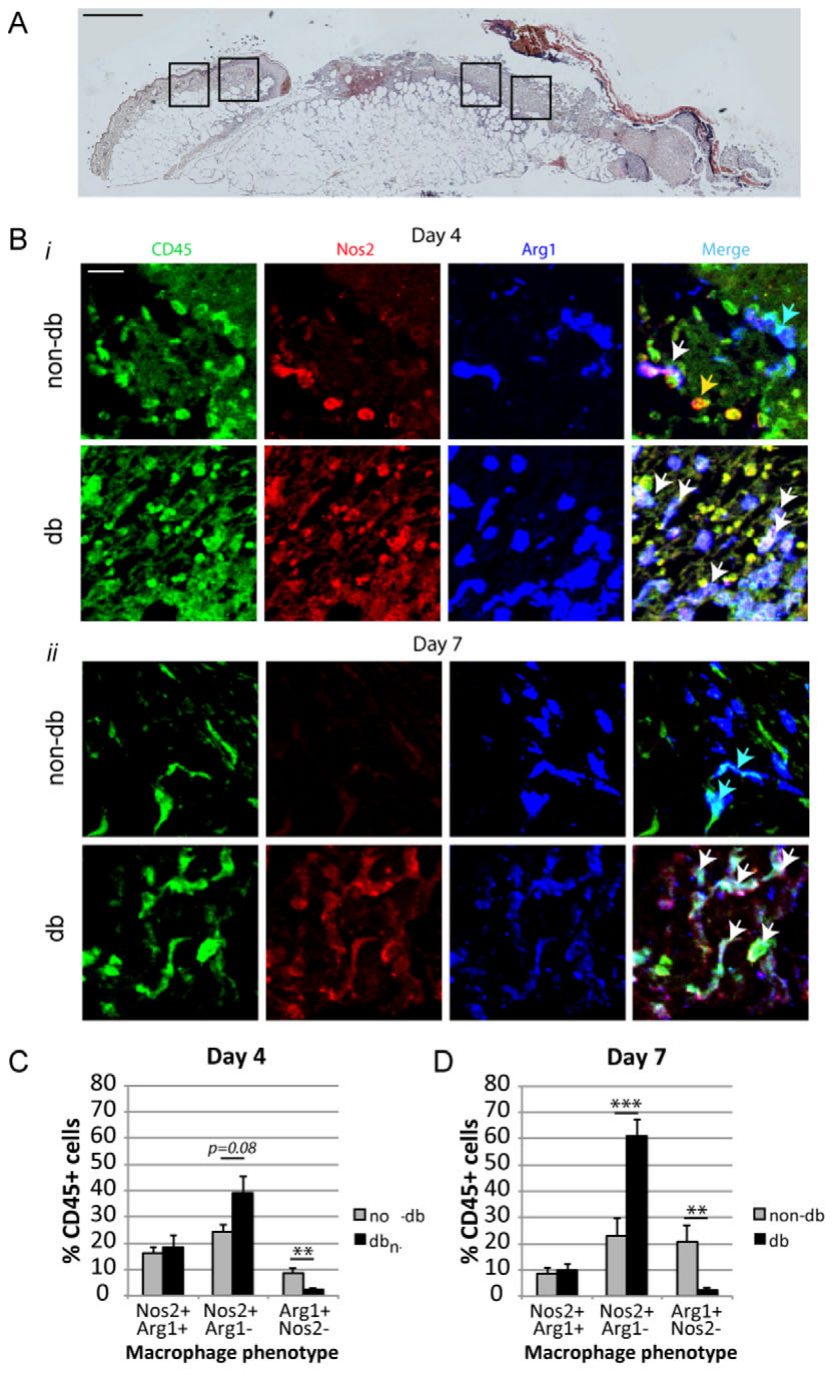
**Analysis of inflammatory cell polarisation in wounds of non-diabetic and diabetic mice over a healing time-course**. (A) Overview of a representative whole-wound section from a day-7 diabetic wound with boxes indicating where images were captured for analyses (scale bar: 1 mm). (B) Immunofluorescent detection of CD45, Nos2 and Arg1 in day-4 (i) and day-7 (ii) wounds of non-db and db mice. Merged images show individual CD45^+^ cells that are also positive for Nos2 but not Arg1 (M1 phenotype, yellow arrow), Arg1 but not Nos2 (M2 phenotype, blue arrow), and for both Nos2 and Arg1 (‘mixed’ phenotype, white arrows) (scale bar: 10 μm). (C,D) Quantification of macrophage phenotypes as shown in B at (C) day 4 following wounding and (D) day 7 following wounding, from six non-db and six db mice at each time point. Non-db, non-diabetic; db, diabetic; **P<0.01, ***P<0.001, unless otherwise indicated.

In normally healing wounds, inflammatory cells initially display both M1 and M2 activation phenotypes during the inflammatory phase (first few days), then switch to a predominantly M2 activation phenotype during the proliferative/neovascularisation phase of healing (by day 7) (Daley et al., 2010). The effect of the diabetic environment on inflammatory cell activation has also recently been examined (Miao et al., 2012; Mirza and Koh, 2011). However, these studies fail to address the phenotype of individual inflammatory cells *in situ.* Therefore, in order to assess the *in situ* phenotype of inflammatory cells in the normal and diabetic wound environment, we triple-labelled wound sections from diabetic (db) and non-diabetic (non-db) mice with antibodies detecting CD45 (pan-inflammatory cell marker), Nos2 (M1 activation marker) and Arg1 (M2 activation marker) in full-thickness excisional wounds. Analyses were performed in the peri-wound dermis and granulation tissue as indicated in Fig. 1A at day 4 (Fig. 1Bi) and day 7 (Fig. 1Bii). Negative control stains for these analyses are presented in supplementary material Fig. S1.

Early in healing (day 4), a relatively large percentage (∼20%) of CD45^+^ cells in both non-db and db wounds were double positive for both Nos2 and Arg1, suggesting a mixed polarisation phenotype, whereas CD45^+^ Nos2^+^ Arg1^−^ inflammatory cells might be more prevalent in db wounds (*P*<0.08). Moreover, CD45^+^ Nos2^−^ Arg1^+^ cells were significantly decreased in db wounds compared with nondb at this time point, suggesting an early skew towards an M1 environment. At a later time point (day 7), the number of CD45, Nos2, Arg1 triple-positive cells was decreased by ∼50% in both nondb and db wounds compared with the day 4 wounds and, although the number of CD45, Nos2 double-positive cells markedly increased in db wounds, it remained the same in non-db wounds (Fig. 1C,D). Additionally, wounds from non-db mice showed a doubling in the number of CD45, Arg1 double-positive cells by day 7, whereas, in db wounds, the number of CD45, Arg1 double-positive cells remained low. At day 7, when wounds of non-db mice have transitioned to a pro-healing phenotype and display significantly less M1-marker inflammatory cells and significantly more M2-marker inflammatory cells, wounds of db animals had dramatically skewed towards an M1 phenotype (Fig. 1D).

In order to further quantify the levels of these M1 and M2 markers in whole wounds over time between non-db and db mice, *Nos2* and *Arg1* qRT-PCR was performed on RNA extracted from whole wounds across a healing time-course. Interestingly, both *Nos2* and *Arg1* showed a similar pattern of expression in non-db mice, with levels peaking for both genes at day 7 following wounding, and declining by day 14 to unwounded levels. However, db mice showed a different profile, with significantly higher expression of both *Nos2* and *Arg1* at day 4, suggesting an early hyperpolarisation phenotype (supplementary material Fig. S1B,C). By day 7, *Nos2* levels in wounds of db mice were ∼tenfold higher compared with non-db wounds, whereas *Arg1* expression began a premature decline, instead of continuing to rise as in the non-db wounds. At day 14, *Nos2* levels remained over tenfold higher than in non-db wounds but, surprisingly, *Arg1* levels, although reduced compared with days 4 and 7, were still elevated compared with non-db wound levels. Because the expression data seemed to contradict the antibody *in situ* analyses, we sought to get an estimate of the relative amounts of *Arg1* to *Nos2* expression at each time point. Early in healing (day 4) the *Arg1:Nos2* ratio indicates that the balance of M1/M2 polarised cells is similar in both non-db and db wounds. However, by day 7 there is a dramatic difference in the *Arg1:Nos2* ratios in non-db and db wounds (supplementary material Fig. S1D). By this time point, the *Arg1:Nos2* ratio has dramatically dropped in db wounds, indicating a pronounced shift towards a pro-inflammatory environment in db wounds. Moreover, by day 14 the *Arg1:Nos2* ratio shows a clear shift towards M2 polarisation in nondb wounds, which is lacking in db wounds. This data clearly supports the antibody analyses in Fig. 1; thus, we suspect the differences seen between the individual gene comparisons and the antibody-derived data might reflect *Nos2* and *Arg1* gene expression by additional cell types in the wound. To assess whether these differences in expression might be related to differences in macrophage differentiation, we analysed whole-wound expression of general macrophage differentiation markers. Expression of *Itgam* (CD11b), *Emr1* (F4.80), *CD68* and *CD14* in day 7 whole-wound samples indicates that general macrophage marker expression is relatively normal in this model (supplementary material Fig. S1).

### The diabetic environment alters recruitment and/or retention of specific monocyte and macrophage subsets

**Fig. 2.**
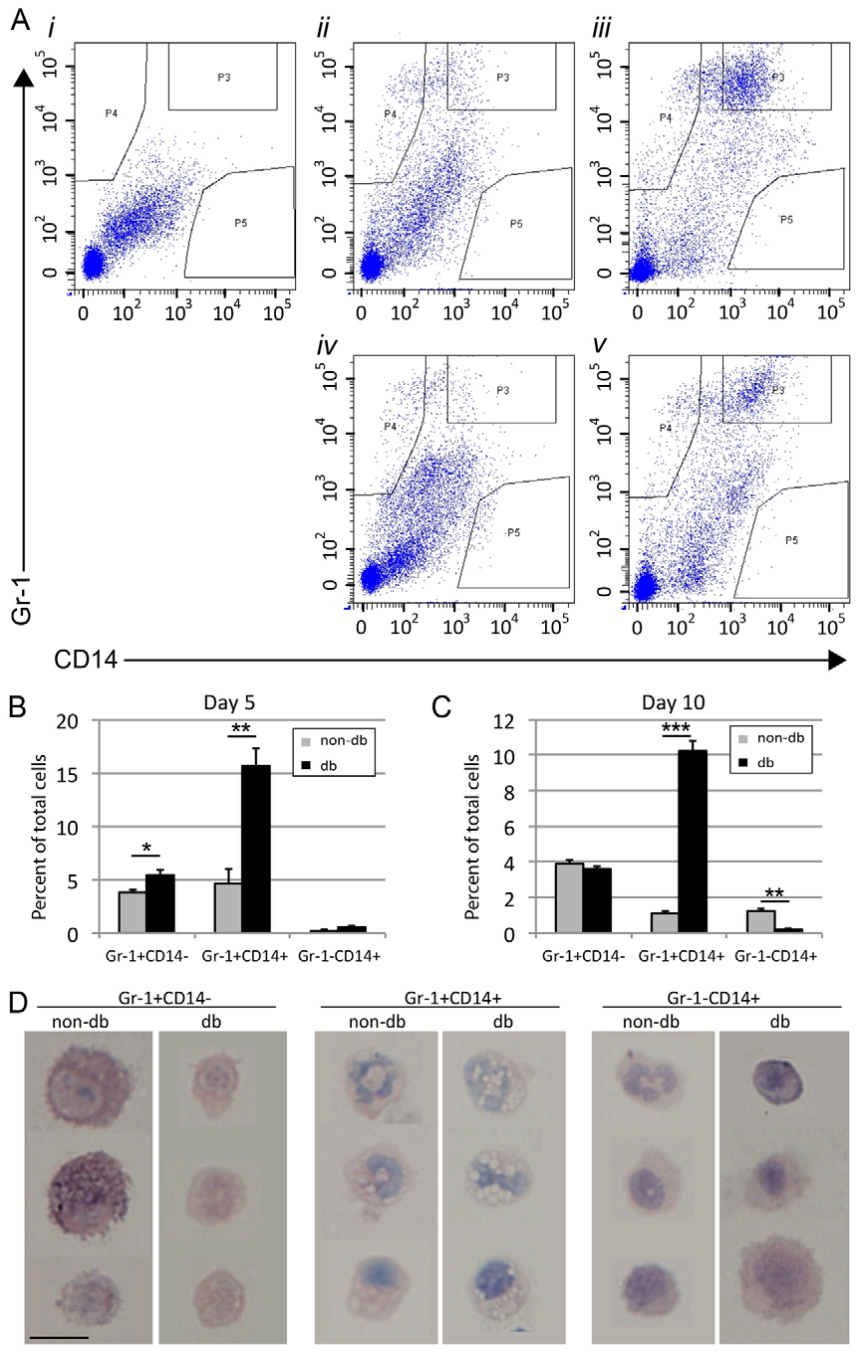
**Analysis of myeloid cell subsets in day-5 and day-10 non-db and db wounds.** (A) Representative flow plots from tissue-dissociated single-cell suspensions of whole wounds gated on CD11b^+^ cells and showing Gr-1 and CD14 expression in (i) isotype control, (ii) non-db day-5, (iii) db day-5, (iv) non-db day-10 and (v) db day-10 wounds. Upper left region of plot (P4) shows cells counted as positive for Gr-1, but negative for CD14. Upper right region of plot (P3) shows cells double positive for Gr-1 and CD14. Lower right region of plot (P5) shows cells negative for Gr-1 but positive for CD14. (B,C) Graphs of flow cytometry data shown in A for day-5 (B) and day-10 (C) wounds displaying percent of total wound cells positive for the listed marker combinations (grey bars, non-db; black bars, db; *n*=6, *P<0.05, **P<0.01, ***P<0.001). (D) Representative images of non-db and db day-5 wound-derived sorted cells shown in A following cytospin and Giemsa staining: Gr-1^+^ CD14^−^ cells (left-most two panels), Gr-1^+^ CD14^+^ cells (centre panels) and Gr-1^−^ CD14^+^ cells (rightmost two panels). Scale bar: 5 μm.

Our immunohistochemistry and gene expression data suggest that the *in vivo* switch from the M1-to-M2 macrophage phenotype normally occurs around 7 days post-wounding, but fails in a diabetic environment. To confirm this in the inflammatory cells, we isolated myeloid cells from wounds of non-db and db mice at day 5, prior to the M1-to-M2 phenotypic switch, and again at day 10, post-phenotypic switch. Tissue-dissociated, single-cell suspensions of whole wounds were incubated with antibodies against CD11b (common myeloid marker), Gr-1 (marker of granulocyte-monocyte progenitors, granulocytes, and a subset of monocytes and macrophages) and CD14 (monocyte and macrophage marker) to quantify myeloid cell subtypes within the wounds. Flow cytometry analysis revealed that there were only marginal differences in the percentage of Gr-1^+^ CD14^−^ cell populations derived from mid (day 5) and late (day 10) healing time points between non-db and db wounds (Fig. 2A–C; supplementary material Table S1). The wound-isolated Gr-1, CD14 double-positive cell population, however, showed a pronounced increase in db wounds compared with nondb at both time points. Finally, the Gr-1^−^ CD14^+^ cell population, although unchanged at day 5, was surprisingly significantly decreased in db wounds compared with non-db (Fig. 2A–C; supplementary material Table S1).

In order to clarify the identity of these myeloid cell subsets, we analysed their morphology using FACS and cytospin analysis. Populations shown in Fig. 2A were isolated from day-5 wounds and examined after Giemsa staining, and representative cells are shown in Fig. 2D. Gr-1^+^ CD14^−^ cells isolated from non-db wounds show a characteristic granulocyte morphology, whereas their db-derived counterparts appear much smaller and less vacuolated (Fig. 2D, leftmost panels). It is unclear why most of the db-derived granulocytes appear to be of the smaller subtype, although some of this subtype is present in the non-db sample as well (e.g. Fig. 2D, bottom cell, left panel). By contrast, Gr-1^+^ CD14^+^ cells, which have previously been classified as short-lived inflammatory monocytes or macrophages that are essential to the antimicrobial response, producing high levels of pro-inflammatory cytokines (Geissmann et al., 2003; Serbina et al., 2008; Daley et al., 2010; Laurent et al., 2011), appear morphologically similar between the two groups and display characteristics of both polymorphonuclear cells and monocytes (Fig. 2D, centre panels). Finally, the Gr-1^−^ CD14^+^ subset, which is virtually lacking in db wounds, shows characteristics of non-inflammatory monocytes or macrophages (lacking vacuoles) and might represent the M2 ‘pro-repair’ macrophages (Fig. 2D, right-most panels). These clear differences in the type and morphology of myeloid cell subsets that are present in the db wounds compared to controls at both pre- and post-phenotypic switch time points indicate abnormal recruitment, retention and/or differentiation during the early inflammatory phase.

### Macrophages and granulocytes from diabetic wounds show higher levels of both M1 and M2 marker expression early in healing but only M1 in late healing

**Fig. 3.**
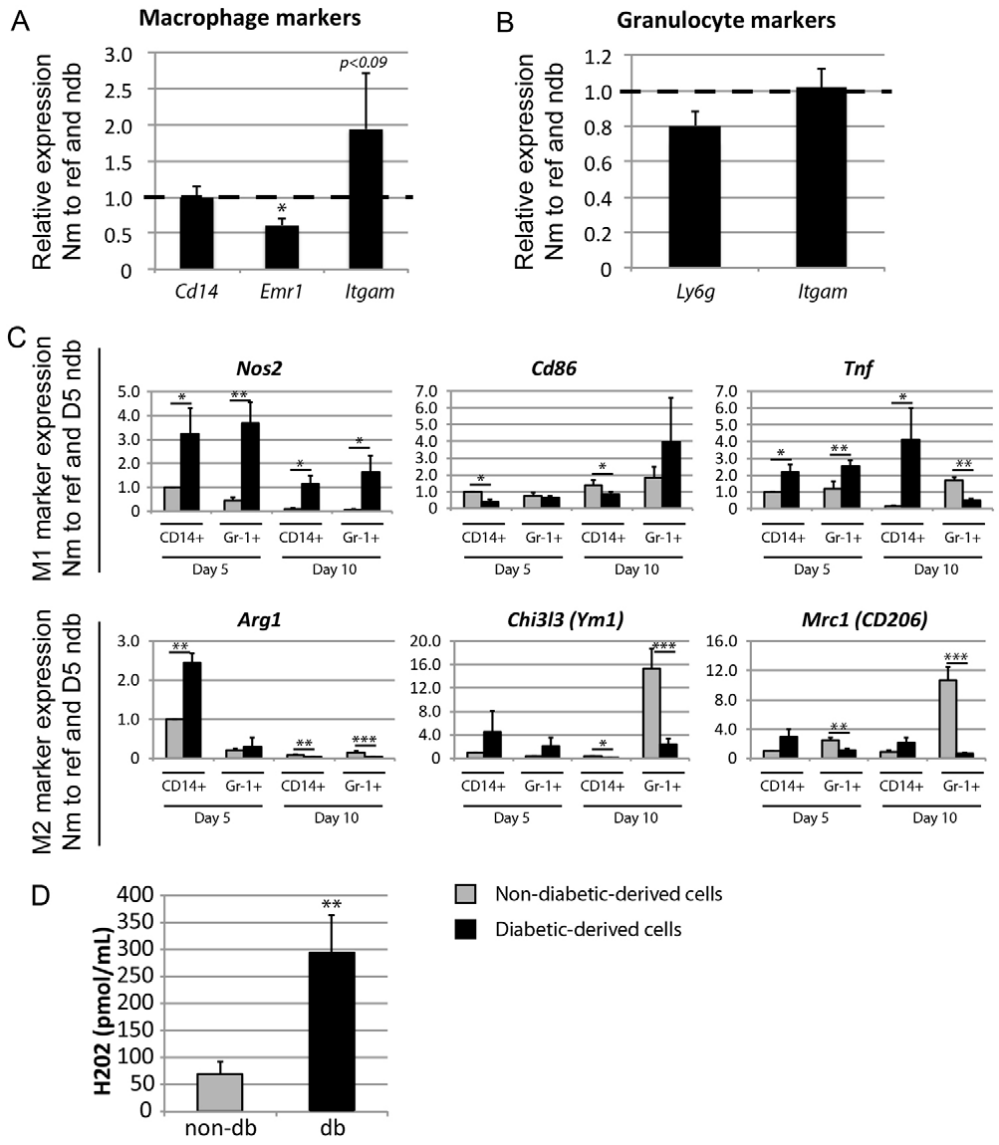
**Analysis of differentiation and polarisation in day-5 and day-10 wound-derived myeloid cells.** (A,B) qRT-PCR analysis of (A) general macrophage maturation marker expression in wound-derived CD14^+^ (all) cells, or (B) general granulocyte maturation marker expression in wound-derived Gr-1^+^ CD14^−^ cells, pooled from three to six non-db or three to six db samples (three pools each) 5 days post-wounding. Bars indicate gene expression in db-derived CD14^+^ (all) cells relative to the non-db sample (indicated by dashed line) and reference genes *Hist2h2aa1* and *Hsp90ab1*. Means and s.e.m. of three biological replicates are shown (*P<0.05). Nm to ref and ndb, normalised to reference genes and non-diabetic samples. (C) Analysis of M1 and M2 polarisation markers in CD14^+^ (all) cells (CD14+) and Gr-1^+^ CD14^−^ cells (Gr-1+) isolated from wounds of non-diabetic (grey bars) or diabetic (black bars) mice 5 or 10 days after wounding (time point indicated, *P<0.05, **P<0.01, ***P<0.001). (D) Analysis of H_2_O_2_ production from wound-derived CD11b^+^ myeloid cells from three non-db and three db mice after 24-hour culture (mean ± s.e.m., **P<0.01).

In order to determine whether myeloid cells derived from db wounds were indeed hyperpolarised *in vivo*, as the whole-wound data suggested, we isolated RNA from db- and non-db-derived CD14^+^ and Gr-1^+^ myeloid cell subsets as shown in Fig. 2. We initially analysed whether there were any apparent differences in macrophage or granulocyte maturation by measuring levels of *CD14*, *Emr1* (F4.80) and *Itgam* (CD11b) in macrophages, and *Ly6g* and *Itgam* in granulocytes. As has been previously reported, macrophages derived from db wounds expressed significantly less *Emr1* (*P*<0.05) and potentially more *Itgam* (*P*<0.09) compared with controls (Fig. 3A), suggesting a less mature macrophage phenotype in the db wound (Bronte et al., 2000; Hirsch et al., 1981), whereas no significant differences were found in the Gr-1^+^ CD14^−^ granulocyte subset (Fig. 3B). In addition, mirroring the whole-wound results, both macrophages and granulocytes from day-5 db wounds showed elevated expression levels of both M1 and M2 marker genes compared with non-db-derived macrophages (Fig. 3C). It would thus seem that db-derived macrophages are capable of developing into the M2 phenotype initially. Notably, db-derived macrophages and granulocytes from a later wound time point (day 10) were hyperpolarised towards the M1 phenotype, with significantly reduced levels of M2 polarisation markers compared with non-db counterparts (Fig. 3C).

These data suggest that myeloid cells recruited to early wounds are intrinsically primed towards a pro-inflammatory phenotype. Production of reactive oxygen species is another indicator of a pro-inflammatory phenotype. Therefore, to further confirm that these cells are intrinsically pro-inflammatory, we cultured CD11b^+^ cells isolated from early wounds of non-db and db mice, removing them from the wound milieu for 24 hours, and measured production of hydrogen peroxide. As shown in Fig. 3D, db-derived myeloid cells produce significantly higher levels of H_2_O_2_ than non-db-derived cells. Our data also supports the idea that myeloid cells recruited to late wounds fail to develop into M2 macrophages as the result of aberrant extrinsic factors present in the local wound environment rather than an intrinsic defect in the ability to adopt an M2 phenotype. However, in order to test this hypothesis more thoroughly, we moved to a cell culture model of macrophage activation.

### Diabetic-derived macrophages are intrinsically pro-inflammatory and hypersensitive to both classical and alternative activation

**Fig. 4.**
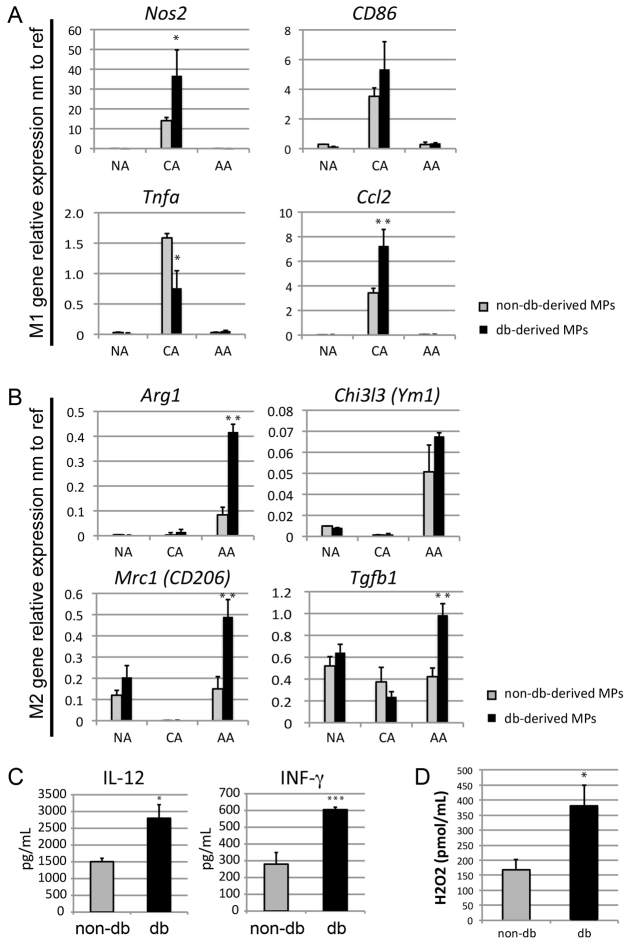
**Analysis of macrophage polarisation markers in BM macrophages after 7 days of culture in M-CSF.** (A,B) Analysis of M1 (A) and M2 (B) polarisation markers in non-activated (NA), classically activated (CA) and alternatively activated (AA) non-db (grey bars) and db (black bars) BM macrophages (*n*=3 independent RNA samples, *P<0.05, **P<0.01). nm to ref, normalised to reference genes. (C) Production of IL-12 and INF-γ in non-db (grey bars) and db (black bars) BM macrophages (*n*=3 independent samples, *P<0.05, ***P<0.001). (D) Analysis of H_2_O_2_ production from BM-derived macrophages from non-db and db mice (*n*=3 independent samples, *P<0.05).

In order to determine whether db-derived macrophages are intrinsically primed to hyperpolarise towards M1 and M2 phenotypes, we assessed expression of M1 and M2 polarisation markers in unstimulated and stimulated BM-derived macrophages (both classical and alternative) from non-db and db mice after 7 days culture in identical environments. Db-derived macrophages showed more extreme polarisation towards an M1 phenotype when classically stimulated, as expected (Fig. 4A), and, consistent with early-wound-derived myeloid cells, also showed an elevated M2 polarisation response when stimulated with alternative-activation-inducing factors (Fig. 4B). Thus, db-derived macrophages seem to be intrinsically primed to hyperpolarise towards either the M1 phenotype or the M2 phenotype.

To determine whether diabetic-derived BM macrophages were intrinsically more pro-inflammatory, non-db and db-derived BM macrophages were assessed for production of pro-inflammatory and anti-inflammatory cytokines. Production of tumour necrosis factor (TNF), IL-6 and IL-10 was very low in both groups (undetectable with ELISA) and did not show significant differences between unstimulated non-db and db macrophages (not shown). However, production of IL-12 and INF-γ was significantly increased in db-derived macrophages compared with non-db equivalent cells (Fig. 4C). Moreover, production of H_2_O_2_ was significantly higher in db-derived macrophages compared with non-db (Fig. 4D). Altogether, these data suggest that db-derived macrophages are intrinsically more pro-inflammatory, independent of environment. Such intrinsic differences could result in aberrant responses to environmental signals and promote pro-inflammatory behaviour during tissue repair and regeneration.

### Diabetic-derived macrophages display normal *in vitro* differentiation, but have aberrant chemotaxis, migration and adhesion

**Fig. 5.**
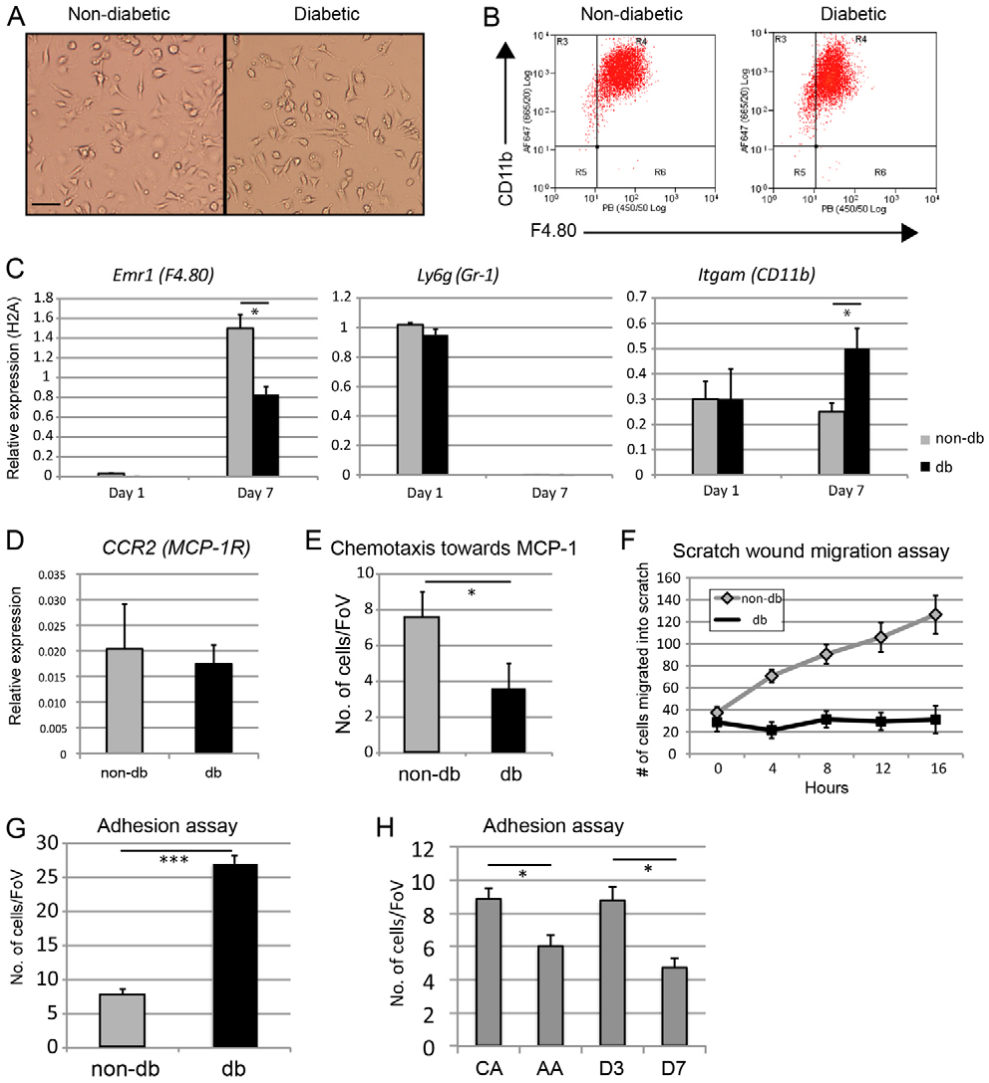
**Differentiation and behavioural assays of non-db- and db-derived BM macrophages.** (A) Representative bright-field images of nondb- and db-derived BM macrophages after 7 days culture in M-CSF (scale bar: 25 μm). (B) Representative flow cytometry plots of non-dband db-derived BM macrophages after 7 days culture in M-CSF, showing expression of macrophage markers CD11b and F4.80. (C) qRTPCR analysis of myeloid cell markers *Emr1* (F4.80, left panel), *Ly6g* (Gr-1, centre panel) and *Itgam* (CD11b, right panel) at day 1 and day 7 of culture in M-CSF in non-db (grey bars) and db (black bars) BM macrophages from three independent RNA isolations for each condition. (D) qRT-PCR analysis of *CCR2* expression in nondb (grey bar) and db (black bar) BM macrophages after 7 days of culture in M-CSF from three independent RNA isolations for each condition. (E) Chemotactic response to MCP-1 in a transwell assay showing mean number of cells per field of view (FoV) of non-db and db BM macrophages from three independent experiments. (F) Scratch wound migration assay of non-db (grey diamonds) and db (black squares) BM macrophages showing mean number of cells migrated into scratch wound at 4-hour intervals over 16 hours from three independent experiments. (G) Adhesion assay of non-db (grey bar) and db (black bar) BM macrophages showing mean number of cells per field of view adhered onto activated endothelial cells after 4 hours from three independent experiments. (H) Adhesion assays comparing classically activated (CA) and alternatively activated (AA) BM macrophages with wound-derived macrophages at days 3 and 7 following wounding (*n*=3 for each condition).

We have previously demonstrated that immature myeloid cells obtained from diabetic mice differentiate more frequently into monocytes rather than to granulocytes compared with those from non-diabetic mice (Mahdipour et al., 2011). To further investigate the influence of diabetes on macrophage differentiation and maturation, we assayed BM-derived cells from db and non-db mice for macrophage differentiation potential and function. Diabetic-derived BM cells showed no apparent gross difference in their differentiation potential (6 day M-CSF stimulation) as assessed by their morphology (Fig. 3A) or immunophenotype, as measured by flow cytometry (Fig. 5B). However, quantification of median fluorescence intensity of CD11b and F4.80 revealed a trend towards reduced CD11b cell surface protein levels (median fluorescence db, 793±85 versus non-db, 1005±165; *P*=0.12) and significantly reduced F4.80 cell surface protein levels (db median fluorescence, 22±7 versus non-db, 53±7; *P*<0.01). Moreover, qRT-PCR analyses revealed that db-derived macrophages expressed significantly less *Emr1* mRNA (encoding F4.80), but significantly more *Itgam* mRNA (encoding CD11b; Fig. 3C) compared with non-db macrophages. The significant reduction in F4.80 was consistent with the wound-derived macrophages, and again might indicate a less mature macrophage phenotype, because *Emr1* expression is restricted to mature macrophages, whereas *Itgam* is expressed on myeloid progenitors as well (Bronte et al., 2000; Hirsch et al., 1981). The discrepancy between *Itgam* mRNA expression and cell surface protein levels of CD11b in db-derived macrophages might be due to dysfunctions in protein translation, misfolding and subsequent degradation, or in localisation in db-derived cells, as has been seen for other proteins (Hamilton et al., 2013; Yu et al., 2007).

Next we performed *in vitro* assays to analyse wound-induced behaviours, recruitment, retention and migration in db-derived versus non-db-derived macrophages. The wound-induced chemokine monocyte chemotactic protein-1 (MCP-1; encoded by the *Ccl2* gene) is expressed at significantly higher levels in wounds of diabetic mice compared with non-diabetic mice (Wetzler et al., 2000). Interestingly, although db- and non-db-derived macrophages expressed similar levels of the MCP-1 receptor (CCR2; Fig. 3D), they displayed an attenuated chemotactic response to MCP-1 (Fig. 3E). To determine whether this might reflect a more general migratory defect, we performed scratch wound assays and analysed migration into the ‘wound’ zone using live imaging. Diabetic-derived macrophages showed a significant defect in scratch wound closure (Fig. 3F).

Pro-inflammatory, classically activated macrophages have been shown to be more adherent than alternatively activated macrophages in a variety of assays (Gordon, 2003; Vereyken et al., 2011). To determine whether diabetic-derived macrophages might be aberrantly retained in the wound environment because of enhanced adhesive properties, we performed adhesion assays by co-culturing macrophages on activated endothelial cells. Compared with non-db-derived macrophages, db-derived macrophages were significantly more adherent (Fig. 3G). For comparison, we also characterised the adhesive properties of classically and alternatively activated macrophages, as well as macrophages obtained from the early, pro-inflammatory wound environment (day 3) and late, anti-inflammatory environment (day 7) of non-diabetic animals in the same assay. As expected, classically activated and day-3 wound macrophages were significantly more adhesive than alternatively activated and day-7 wound macrophages (Fig. 3H). Taken together, these data suggest that db BM-derived macrophages cultured outside of the diabetic environment retain aberrant differentiation and functional characteristics, suggesting that persistent intrinsic differences might underlie a pro-inflammatory-prone phenotype.

### Diabetic environment, not Lepr deficiency, induces the aberrant macrophage phenotype

One important intrinsic factor that distinguishes db-derived versus non-db-derived macrophages in this mouse model of type 2 diabetes is the hypomorphic allele of the leptin receptor carried by *Lepr*^−/−^ mice. This allele carries a G>T point mutation in the non-coding region of the leptin receptor gene that promotes aberrant splicing of a 106-nucleotide insertion, creating a novel exon in the long isoform of the receptor, resulting in aberrant signalling that might affect immune cell behaviour (Chen et al., 1996; Busso et al., 2002). However, it has been previously shown that leptin signalling in wound-resident cells has no effect on inflammatory cell recruitment and/or retention dynamics, because topical administration of leptin in the leptin-deficient mouse model of diabetes did not rescue the aberrant inflammatory response, whereas systemic administration did reduce neutrophil, but not macrophage, recruitment and/or retention (Goren et al., 2003). To test whether inflammatory cell behaviour with regards to wound recruitment and retention is dependent on inflammatory-cell-intrinsic Lepr signalling, we performed BM transplantation from non-db donors into non-db and db recipients as well as from db donors into non-db and db recipients. Donors were transgenic for ubiquitously expressed eGFP under the control of the chicken β-actin promoter (Okabe et al., 1997) to facilitate tracking of transplanted cells.

**Fig. 6.**
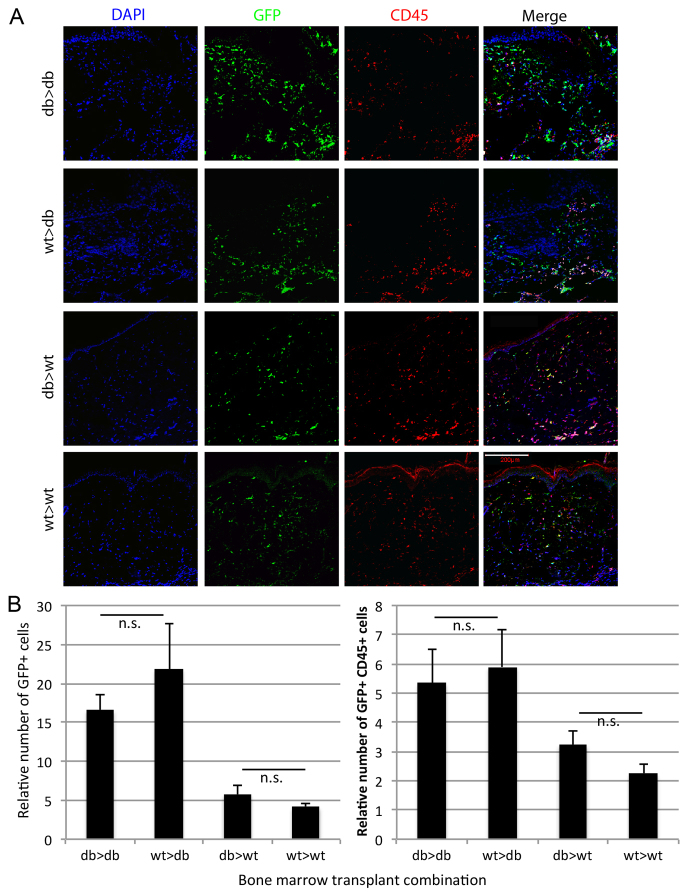
**Analysis of the role of the leptin receptor on recruitment and retention of inflammatory cells in non-diabetic and diabetic wound environments.** (A) Representative images of total cells (DAPI), transplanted *Lepr*^+/−^ (wt) and *Lepr*^−/−^ (db) BM-derived cells (GFP) and inflammatory cells in sections of non-diabetic (wt) and diabetic (db) wounds in the following donor >recipient combinations: *Lepr*^−/−^ donor into *Lepr*^−/−^ recipient (db >db, top row of panels), *Lepr*^+/−^ donor into *Lepr*^−/−^ recipient (wt >db, second row of panels), *Lepr*^−/−^ donor into *Lepr*^+/−^ recipient (db >wt, third row of panels) and *Lepr*^+/−^ donor into *Lepr*^+/−^ recipient (wt >wt, bottom row of panels). Scale bar: 200 μm. (B, left panel) Quantification of recruitment and retention data of total BM-derived cells (GFP positive) in the transplant combinations shown in A (*n*=6; n.s., not significant). (B, left panel) Quantification of recruitment and retention data of BM-derived inflammatory cells (double positive for GFP and CD45) in the transplant combinations shown in A (*n*=6; n.s., not significant).

Mice were wounded 5 weeks after transplant, and wounds harvested at day 7. Consistent with previous studies (Mace et al., 2009), the number of GFP^+^ cells in db recipient wounds was three-to four-fold higher than non-db recipient wounds (Fig. 6A,B), independent of the donor genotype. Likewise, the number of BM-derived inflammatory cells (GFP^+^ CD45^+^) in db recipient wounds was ∼twofold higher than in non-db recipient wounds (Fig. 6A,C), independent of the donor genotype. We also performed adoptive transfer experiments in which GFP^+^ db-derived BM macrophages were injected into day-3 wounds of non-db and db mice to test whether the wound-resident cells had any involvement in the hyperpolarisation phenotype of recruited myeloid cells *in vivo*. Non-db-derived macrophages injected into non-db wounds were used as a control. We found that db-derived macrophages still showed a hyperpolarisation response with regards to M1 markers, but showed a mixed response (both hyper- and hypo-polarisation) with regards to M2 markers, indicating that db-derived macrophages are intrinsically more pro-inflammatory *in vivo* (supplementary material Fig. S2). Interestingly, we did observe a further increase in both Nos2 and CD86 expression when db-derived macrophages were transferred to the db wound environment; thus, it is likely that extrinsic factors also play a role in macrophage phenotype.

Finally, to test the role of the leptin receptor in aberrant macrophage polarisation, we obtained *Lepr*^−/−^ macrophages from 3-week-old pups, prior to the onset of diabetes. Following classical and alternative activation stimuli, we found that there was no difference in the response of these macrophages compared with 3-week-old non-db control macrophages (supplementary material Fig. S3). Altogether, these data clearly show minimal involvement of inflammatory-cell-intrinsic leptin receptor function in wound recruitment and/or retention dynamics of inflammatory cells, and support the idea that the diabetic environment induces prolonged, intrinsic changes in myeloid cells that result in pro-inflammatory priming.

### Human non-healing wounds are also characterised by reduced M2 macrophage polarisation

**Fig. 7.**
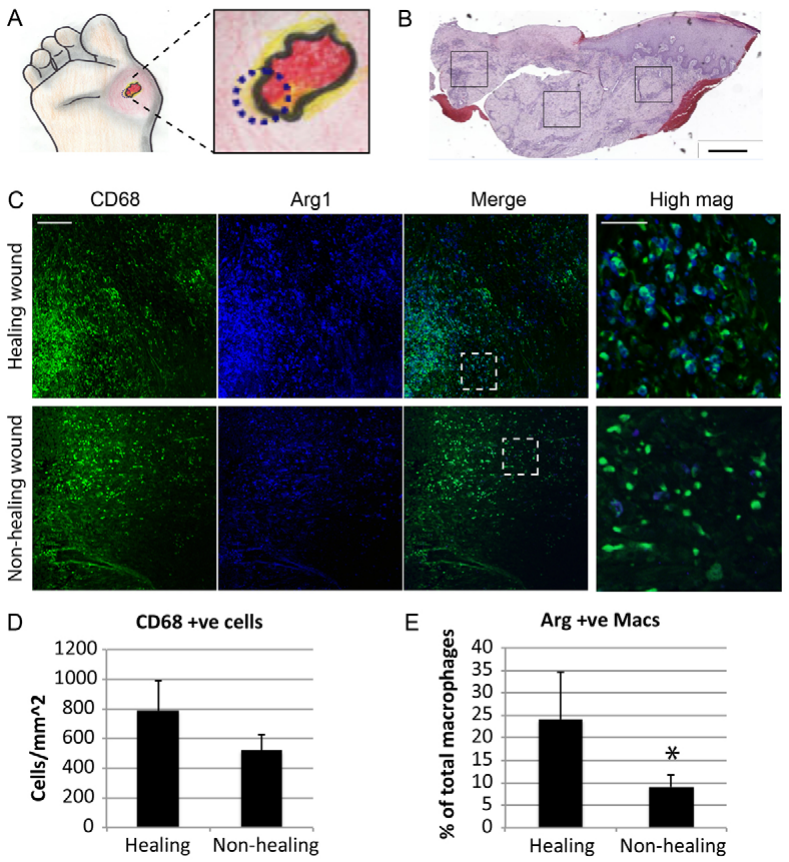
**Analysis of Arg1^+^ cells in human healing and non-healing diabetic foot ulcers.** (A) Cartoon diagram showing punch biopsy site, indicated by blue dotted circle. (B) H&E stain of whole-wound section showing representative areas used for analyses of CD68, Arg1 and Nos2 immunofluorescent staining (scale bar: 600 μm). (C) Representative images of CD68 and Arg1 immunofluorescent staining in sections from healing and non-healing diabetic foot ulcers. The first three panels of each group show fluorescent staining as indicated (scale bar: 100 μm). The fourth panel of each group shows a high-magnification view of the area indicated by the white box in the merge panels (scale bar: 25 μm). (D) Quantification of the number of CD68^+^ cells per mm^2^ in healing and non-healing wounds. (E) Quantification of the percentage of macrophages (Macs) that are Arg1^+^ (double positive for Arg1 and CD68) in healing and non-healing wounds (*n*=7, *P<0.05).

We next examined human diabetic wounds to determine whether a failure of M2 polarisation would directly correlate with a failure to heal. Specifically, we obtained biopsy tissue from the wound margin of human diabetic foot ulcers at presentation (Fig. 7A). Patients then received current best practice care (including wound offloading) and were monitored for a period of 6 weeks. At the end of this period wounds were segregated into those that healed and those that remained unhealed (non-healing). Immunofluorescent quantification of wound biopsy tissue sections in fields of view from the dermis and granulation tissue (Fig. 7B) revealed similar numbers of macrophages (CD68^+^ cells; Fig. 7C,D) but a pronounced reduction in CD68, Arg1 double-positive cells in ulcers that failed to heal compared with those that healed (Fig. 7C,E). Negative control stains are shown in supplementary material Fig. S4A. Interestingly, there was no difference in CD68, Nos2 double-positive cells (supplementary material Fig. S4B,C). These data suggest a clear link between M2 polarisation and wound healing efficacy, and support the concept that M2 polarisation in diabetic wounds is a crucial factor in healing outcome.

## DISCUSSION

Studies using a variety of animal models, and also patient samples, have revealed that the diabetic environment negatively affects wound healing. Over the past two decades it has become clear that reduced recruitment of progenitor cells combined with an over-abundance of inflammatory cells contributes to the development of chronic wounds. Indeed, many extrinsic factors have been identified that play a role in this pathological condition, including reduced levels of growth factors, such as PDGF, TGF-β, VEGF, KGF and FGF2 (Beer et al., 1997; Frank et al., 1995; Frank et al., 1996; Greenhalgh et al., 1990; Werner et al., 1994), and overexpression of pro-inflammatory factors, such as TNF, MIP-2 and IL-1 (Wetzler et al., 2000).

Many of these extrinsic factors are initially produced by wound-resident cells, then later by recruited inflammatory cells, acting in an autocrine/paracrine manner to alter the healing trajectory. However, the number of inflammatory-cell-intrinsic factors that are reported to be deregulated by the diabetic environment is very low. Notably, Hoxa3 and Hif-1α are two examples of pro-angiogenic transcription factors expressed by both wound-resident and recruited inflammatory cells (Mace et al., 2005; Mace et al., 2007). It remains unclear whether the lack of identified intrinsic factors is due to a lack of focused attempts or because extrinsic factors do indeed play a much larger role in dictating the behaviour of inflammatory cells.

We have begun to address this gap in knowledge by first asking whether intrinsic factors play a role in controlling the phenotype of macrophages during wound healing and, if so, at what phase of the wound healing process are they most likely to be influential. In this study we report that cell-intrinsic factors might play a large role in establishing an initial pro-inflammatory polarisation of recruited inflammatory cells. Our results clearly demonstrate that BM stem and progenitor cells cultured outside of the diabetic environment in identical conditions to non-db-derived BM cells differentiate into macrophages that are significantly more pro-inflammatory than their non-db counterparts. This was demonstrated by both gene expression signatures and behavioural assays. One limitation of this study was the sole use of the leptin-receptor-deficient model of diabetes. However, we have ruled out any role for inflammatory-cell-intrinsic function of the leptin receptor in macrophage recruitment and/or retention dynamics, or polarization. There might be other differences, though, that are specific to this model. Unsurprisingly, these db-derived macrophages respond to classical activation stimuli (LPS + INF-γ) by hyperpolarising towards the M1 macrophage phenotype. However, what was surprising was the finding that these cells also hyperpolarise towards an M2 macrophage phenotype in response to alternative stimuli (IL-4 + anti-INF-γ) *in vitro*, and at early wound time points *in vivo*. It is not until the later wound time points that recruited macrophages fail to develop into M2 macrophages. This important difference clearly demonstrates that db-derived macrophages can readily develop into M2 ‘pro-healing’ macrophages, and that cell-intrinsic factors pre-prime these cells for hyperpolarisation to both pro- and anti-inflammatory stimuli. Crucially, extrinsic factors in the db wound environment direct their prolonged M1 polarisation, resulting in macrophages that show a mixed phenotype (both M1 and M2 markers simultaneously) at later stages of wound healing, when the non-diabetic wound is comprised of predominantly M2 macrophages.

Recent studies suggest that CCR2^+^ M1/M2 mixed-phenotype myeloid cells make up the majority of pro-angiogenic macrophages during wound healing and, consistent with our data, are present at early wound time points (Willenborg et al., 2012). It is puzzling then, why these cells, although clearly present in db wounds, fail to stimulate angiogenesis, a crucial component of healing. Similar analyses of a related myeloid cell population showed that these cells, although normally pro-angiogenic, also failed to stimulate angiogenesis owing to aberrant intrinsic factors (Mahdipour et al., 2011). One explanation is that recruited macrophages fail to mature properly in the diabetic environment and thus fail to initiate an appropriate pro-angiogenic programme. This might be due to differences in epigenetic chromatin regulation, restricting gene expression to a pro-inflammatory programme and/or preventing phenotypic switching. Pro-inflammatory phenotypes due to aberrant epigenetic chromatin regulation have been recently reported in diabetic vascular smooth muscle cells (Villeneuve et al., 2008).

It is not yet clear whether the initial excessive inflammatory response, i.e. the increased numbers of inflammatory cells at the wound site as well as the hyperpolarisation of db M1 macrophages, ‘sets the stage’ for the pro-inflammatory environment in db wounds. The mechanisms underlying altered recruitment and/or retention of inflammatory cells in diabetic wounds have yet to be elucidated. It is interesting to note that diabetic-derived macrophages have reduced chemotaxis to MCP-1, yet accumulate in wounds at much higher numbers by day 7. This might be due to increased retention rather than recruitment; however, this has yet to be determined. Hyperpolarisation might provide a positive feedback loop that sustains the M1 inflammatory response. Alternatively, other cell types in the wound, such as vascular endothelial cells, might play a major role in directing macrophage behaviour. Interactions between macrophages and endothelial cells might be altered in the diabetic environment and affect recruitment kinetics of inflammatory cells. Further investigations into exactly which cell-intrinsic factors are causative of this pre-primed pro-inflammatory phenotype, as well as which cell types in the wound environment contribute to the sustained M1 macrophage phenotype, are necessary and have clear implications for future therapies aimed at promoting healing in individuals with diabetes.

## MATERIALS AND METHODS

### Animals

All animals used in this study were housed at the University of Manchester animal care facility. All procedures were approved by the local ethical review committee and the Home Office. *Lepr^db/db^* and *Lepr^db/+^* mice were purchased from the Jackson Laboratory (Bar Harbor, ME) and Harlan (Oxfordshire, UK). Heterozygous animals were crossed to the C57BL/6-Tg(CAG-EGFP)1Osb/J line, which ubiquitously express enhanced GFP (Jackson Labs), and subsequently backcrossed to produce *Lepr^db/db^* animals carrying the eGFP transgene for BM transplant experiments. All animals were used between 8 and 16 weeks of age, and were age and sex-matched to controls.

### Wounding model

Diabetic and non-diabetic control animals were anaesthetized and the dorsum shaved and sterilized with antiseptic wipes. Full thickness wounds of 8 mm diameter were excised, including the panniculus carnosus layer. Animals received buprenorphine at the time of surgery and were housed in separate cages until tissue was harvested at the described time points by removing the entire wound area, including a 2 mm perimeter.

### Wound processing for immunohistochemistry

Wounds were harvested from sacrificed animals at the indicated time point, cut in half and fixed in formalin overnight, then embedded in paraffin. Wounds were then cut into 5-μm sections using a Microm HM 330 microtome and stained using haemotoxylin and eosin to locate matching section morphology for further analysis. Sections were then dewaxed and rehydrated, followed by sodium citrate buffer antigen retrieval. Sections were incubated with the following antibodies, in various combinations depending on the experiment, diluted in PBT (PBS + 0.1% Tween-20): rat anti-CD45 (1:50, Invitrogen/Life Technologies, clone 30-F11, MCD4500), goat anti-Arg1 (1:200, Santa Cruz Biotechnology, sc-18354), rabbit anti-Nos2 (1:100, Santa Cruz Biotechnology, sc-651), rabbit anti-GFP (1:1000, Abcam, ab6556), followed by the following secondary antibodies as appropriate: donkey anti-rat Alexa Fluor 488 (1:500, Invitrogen/Life Technologies, A-21208), donkey anti-goat Cy5 (1:500, Abcam, ab6566), donkey anti-rabbit Alexa Fluor 555 (1:500, Invitrogen/Life Technologies, A-31572). Sections were then mounted in Prolong Gold with DAPI (Invitrogen/Life Technologies, P36930) and images captured sequentially on an inverted Olympus FV1000 confocal microscope. Three non-db and three db animals were used for each time point. Four fields of view from each wound were taken from both the peri-wound dermis and the granulation tissue in consistent locations from wound to wound. Images were then analysed to determine the number of CD45^+^ Nos2^+^ Arg1^+^, CD45^+^ Nos2^+^ Arg1^−^ and CD45^+^ Nos2^−^ Arg1^+^ cells in each sample using Photoshop to determine the marker overlap. The percentage of each (double positive or triple positive) was calculated and is reported as the mean ± s.e.m. of all images for each category (*n*=12 for each).

### Cell isolation from wounds and flow cytometry

Myeloid cells were isolated from wounds from non-db and db mice at day 5 and day 10 following wounding on three separate occasions, and cells of each subtype (CD14^+^ Gr-1^+^, CD14^+^ Gr-1^−^ or CD14^−^ Gr-1^+^) were pooled together from three to six mice on each occasion and used for cytospin analyses and RNA isolation. Tissue dissociation of wounds was performed essentially as described previously (Mahdipour et al., 2011) with modifications. In brief, freshly harvested wound tissue was weighed and cut into 2-mm pieces followed by overnight incubation at 4°C in 20 μl/mg wound tissue of HBSS containing 1 mg/ml dispase I (Sigma), 3% FBS and 10 mg/ml G418 (Sigma). Tissue was then transferred to HBSS (80 μl/mg of tissue) containing 1 mg/ml collagenase D (Roche), 75 U/ml DNase I (Qiagen) and 5 mg/ml G418 for 2 hours at 37°C in a shaking incubator. After 2 hours the cell suspensions from both steps were mixed and passed through a 70-μm cell strainer (BD Biosciences), counted, centrifuged and washed three times with PBS + 3% FBS, then resuspended in PBS + 3% FBS for antibody incubation. Cells were blocked with Fc block for 5 minutes at room temperature and then incubated with PE-Cy7-conjugated CD11b, APC-conjugated anti-Gr-1 and FITC-conjugated anti-CD14 (all eBiosciences) for 1 hour at 4°C, washed 3× in PBS + 3% FBS, and analysed and/or sorted on a BD FACS Aria (BD Biosciences).

### RNA isolation, cDNA synthesis and qRT-PCR

Wounds were harvested as described above, weighed, snap-frozen on dry ice and stored at −80°C. Samples were then homogenised on ice in 1 ml Trizol (Invitrogen) per 200 mg tissue. Cells isolated from FACS were snap-frozen and stored at −80°C, and then homogenised in Trizol according to the manufacturer’s instructions. RNA from all samples was quantified using a Nanodrop 1000 spectrometer (Thermo), and stored at −80°C. cDNA was synthesised using 0.1-1 μg RNA and BioLine reverse transcriptase reagents according to the manufacturer’s instructions. qRT-PCR was performed on a StepOnePlus (Applied Biosystems) using Taqman Fast Universal Master Mix (Applied Biosystems) and the following primer/probe sets: *Arg1* (Mm00475988_m1), *Ccr2* (Mm00438270_m1), *Cd14* (Mm00438094_g1), *Cd68* (Mm03047340_m1), *Cd86* (Mm00444543_m1), *Chi3l3* (Mm00657889_m1), *Emr1* (Mm00802529_m1), *Itgam* (Mm00434455_m1), *Ly6g* (custom-made, F: 5′-GCGTTGCTCTGGAGATAGAAGTTAT-3′, R: 5′-GATGGGAAGGCAGAGATTGCT-3′, P: 5′-GTGGACTCTCACAGAAGCAA-3′), *Mrc1* (Mm01329362_m1), *Nos2* (Mm01309897_m1), *Tgfb1* (Mm01178820_m1) and *Tnf* (Mm00443258_m1) (Applied Biosystems). *Hist2h2aa1* (Mm00501974_s1) and *Hsp90ab1* (Mm00833431_g1) were used as reference genes.

### Culture of BM-derived macrophages

BM was flushed from femurs and tibiae of three non-db and three db mice in DMEM, passed through a 70 μm mesh filter and plated at 10 million to 20 million cells per 10-cm dish in 7 ml macrophage medium (DMEM + 10% FBS + 10% L-929-conditioned medium containing M-CSF) at 37°C with 5% CO_2_ for 7 days. Medium was supplemented with 7 ml fresh medium on day 4. Non-adherent cells were removed at day 7 and images of adherent cells taken on an inverted Olympus IX70 microscope with a Sony DXC-950p power HAD 3CCD colour video camera. RNA was isolated from day-1 or day-7 cultured macrophages, as indicated, from three nondb and three db animals and used for gene expression analyses.

Cells were analysed by flow cytometry using Pacific Blue-conjugated anti-F4.80 and AF-647-conjugated anti-CD11b (both eBiosciences) on a CyAn ADP Analyzer (Beckman Coulter). Macrophages from each mouse were split into three groups and left as non-activated controls, classically activated by supplementing medium with 100 ng/ml INF-γ (Sigma) and 100 ng/ml LPS (Sigma) or alternatively activated by supplementing the medium with 50 μg/ml anti-INF-γ (Bio X Cell) and 20 ng/ml IL-4 (Peprotech) for 24 hours. RNA was isolated independently from each animal and from each group.

### H_2_O_2_ assay

H_2_O_2_ production by BM-derived macrophages and wound-derived myeloid (CD11b^+^) cells was measured using the Hydrogen Peroxide Assay Kit (Abcam) and by following the manufacturer’s guidelines for the fluorometric assay. BM cells were cultured as described above for 6 days and then a subset from six different animals (three non-db and three db) were plated in 24-well plates in DMEM + 10% FBS. Wound-derived cells were isolated using rat anti-CD11b (M1/70 clone, Abcam) labelled with DSB-X-biotin (Molecular Probes), streptavidin-coated magnetic beads (FlowComp Dynabeads, Invitrogen) and an EasySep magnet (StemCell Technologies). Unconditioned medium was used as a control for background fluorescence, which was subtracted from non-db and db myeloid cell conditioned medium samples. Fluorescence (Ex/Em=535/587) was read in black 96-well plates on a Mithras LB 940 plate reader (Berthold Technologies).

### Migration and adhesion assays

For chemotaxis assays, BM- or wound-derived macrophages were starved in serum-free DMEM medium (GIBCO) for 4 hours before use. They were then seeded 10^5^ cells per well into the top of a trans-well 0.8-μm filter insert (Costar) suspended over the chemoattractant MCP-1 (10 ng/ml, R&D Systems) or serum-free medium. The cells were incubated at 37°C for 1.5 hours before being fixed using ice-cold methanol and stained with Giemsa (Sigma). The inside of the insert was wiped with a cotton swab to remove non-migrated cells. Stained cells on the lower side of the filter were imaged using a Nikon snapshot wide-field microscope and counted (five fields of view/insert). The mean number of cells for each insert was calculated and three replicates were obtained for each condition.

For scratch wound assays, 10^5^ BM-derived macrophages were plated in 24-well plates and allowed to adhere to the plate for 12–16 hours. A scratch in the monolayer was created using a sterile pipette tip, and cells were washed once in PBS. Cells were then incubated in normal culture medium in a climate-controlled microscope box (37°C, 5% CO_2_) and imaged every 10 minutes for 18 hours on an AS MDW live cell imaging system (Leica) using a 10× objective and imaging software Image Pro 6.3 (Media Cybernetics). Point visiting was used to allow multiple positions to be imaged within the same time-course. The images were collected using a Coolsnap HQ CCD camera (Photometrics) and analysed using ImageJ (NIH). A box marking the original scratch was used to calculate the number of cells migrating into the scratch over time. Means of three biological replicates were then plotted using Microsoft Excel.

For adhesion assays, the endothelial cell line bEnd5 (Wagner and Risau, 1994), a kind gift from Dr Charlotte Allen (University of Manchester), were plated to ∼100% confluency by seeding 10^5^ cells per well in a 24-well plate (Costar). Cells were activated using 10 ng/ml TNF (BD Biosciences). BM- or wound-derived macrophages were counted, stained with PKH26 (Sigma) for 10 minutes and then 2×10^4^ cells were added to each well containing the endothelial cells. The cells were incubated at 37°C for 4 hours to allow the cells to adhere before washing with PBS to remove the non-adhered cells. The cells were fixed using 4% paraformaldehyde and imaged using a DM16000B inverted fluorescent microscope (Leica). Ten fields of view were averaged for three biological replicates.

### ELISA

ELISAs for TNF, IL-6, IL-10, INF-γ and IL-12 were performed according to the kit manufacturer’s instructions (Ebiosciences). In brief, plates were coated with 100 μl/well of capture antibody, washed and samples or standards added to each well followed by incubation at room temperature for 2 hours. Wells were then washed and detection antibody added for 1 hour at room temperature. Wells were washed again and avidin-HRP added for 1 hour at room temperature. Wells were washed again and substrate solution was then added to each well and incubated for 15 minutes at room temperature. Finally, stop solution (1 N H_2_SO_4_) was added to each well to stop the colour change reaction. The plate was then read on a plate reader at the appropriate wavelength according to the manufacturer’s instructions (Berthold Technologies, Germany).

### BM chimaeras

BM was harvested from either db or non-db donor mice ubiquitously expressing enhanced GFP by flushing the femurs and tibiae with DMEM. BM was passed through a sterile 27-gauge needle and through a 70-μm nylon mesh cell strainer (BD Biosciences), rinsed once in PBS, and viable cells counted. Chimaeras were generated by injecting 10^6^ viable cells into the tail vein of lethally irradiated (10 Gy) mice within 6 hours following irradiation. Mice were maintained on antibiotic-treated water (sulfamethoxazole/trimethoprim ‘Ditrim’, 5 ml/200 ml) for 2 weeks and allowed 5 weeks for reconstitution before any further procedures were performed.

### Adoptive transfer experiments

On two separate occasions, BM macrophages were prepared and cultured as described above from non-db and db GFP^+^ mice. After 7 days of culture, 4×10^5^ cells were injected into day-3 wounds of one non-db and two db mice in a total volume of 200 μl as follows: non-db macrophages (MP) >non-db wound (wnd), db MP >nondb wnd and db MP >db wnd. After 24 hours, wound tissues were dissociated as described above and GFP^+^ cells sorted on a BD FACS Aria (BD Biosciences). Six independent RNA isolations were performed, cDNA produced, and macrophage marker genes analysed as described above.

### Preparation of macrophages from 3-week-old pups

Three-week-old pups from *Lepr* heterozygous parents were genotyped from ear punch genomic DNA isolations by sequencing PCR products obtained using primers flanking the mutation (*Lepr* F: 5′-CCCTCCCCTCTCCTAAGTGT-3′; R: 5′-CAGCAACCGTCACACCATTA-3′). Blood glucose levels were measured to verify normal levels and BM was then isolated from two *Lepr*[+/−] and two *Lepr*[−/−] pups, cultured, and activated in duplicate for each animal, as described above for adult BM macrophages. Eight independent RNA isolations were performed, cDNA produced, and macrophage marker genes assessed, as described above.

### Human diabetic wound analyses

Local ethical approval was obtained for all human studies. Human diabetic wound samples were obtained from patients at the time of presentation at the Manchester Diabetes Centre following informed consent in accordance with the Declaration of Helsinki. Samples were fixed in 10% formalin, and paraffin embedded. Sections were incubated with the following antibodies: goat anti-Arg1 (Santa Cruz Biotechnologies), mouse anti-CD68 (DAKO), and rabbit anti-Nos2 (Santa Cruz Biotechnologies) followed by incubation with secondary antibodies donkey anti-goat Cy5, donkey anti-mouse Alexa Fluor 488, and donkey anti-rabbit Alexa Fluor 555 for triple labelling. The number of CD68 single-positive cells, Arg1 and CD68 double-positive cells as well as Nos2 and CD68 double-positive cells were counted in five fields of view from healing and non-healing samples (*n*=7 for each), and the mean ± standard deviation plotted.

### Statistical analyses

All results are presented as mean ± s.e.m. unless otherwise noted. Analyses were performed using Microsoft Excel or GraphPad Prism. Means of sample data were compared using ANOVA for three or more groups, followed by post-hoc *t*-tests, or for two groups, a Student’s *t*-test was used. Differences between groups were considered significant at three levels (*P*-values of <0.05, <0.01 and <0.001) and are indicated in the text or within the figure legend.

## Supplementary Material

Supplementary Material
