# Supplementary material for: Diabetes induces stable intrinsic changes to myeloid cells that contribute to chronic inflammation during wound healing in mice
Source: Dis Model Mech. 2013 Sep 18;6(6):1434–47. doi: 10.1242/dmm.012237 (PMC3820266; doi:10.1242/dmm.012237)
Supplement: Supplementary Material [file supp_6_6_1434__index.html]

Diabetes induces stable intrinsic changes to myeloid cells that contribute to chronic inflammation during wound healing in mice — Diabetes induces stable intrinsic changes to myeloid cells that contribute to chronic inflammation during wound healing in mice — Supplementary Material 

# Diabetes induces stable intrinsic changes to myeloid cells that contribute to chronic inflammation during wound healing in mice

## DMM012237 Supplementary Material

**Files in this Data Supplement:**

- **Supplementary Material PDF**
