# Supplementary material for: Diabetes induces stable intrinsic changes to myeloid cells that contribute to chronic inflammation during wound healing in mice
Source: Dis Model Mech. 2013 Sep 18;6(6):1434–47. doi: 10.1242/dmm.012237 (PMC3820266; doi:10.1242/dmm.012237)
Supplement: Supplementary Material [file supp_012237_DMM012237.pdf]

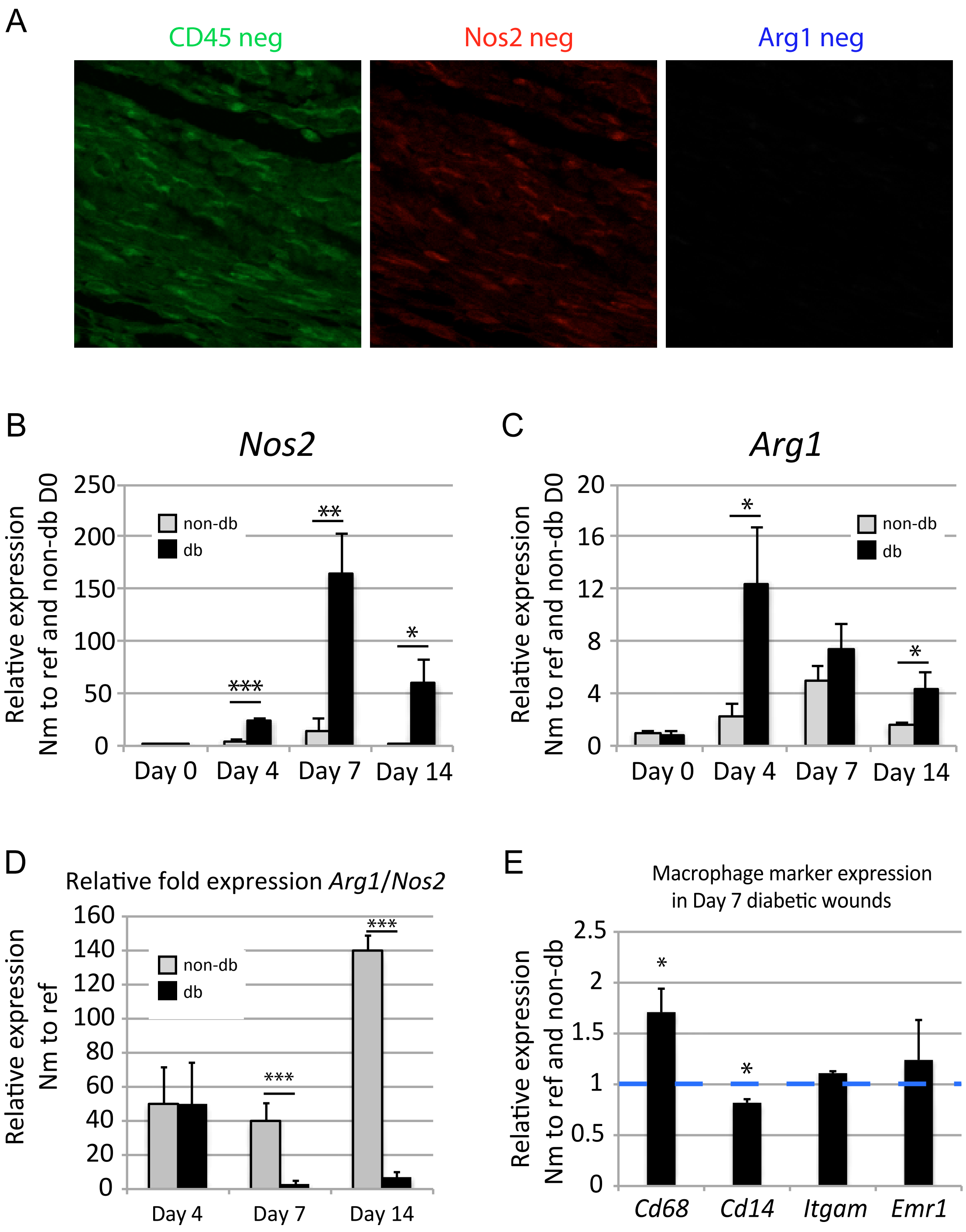


**Supplementary Fig. 1. Whole wound analysis of macrophage polarization and differentiation markers.** (A) Negative controls (secondary only) for immunofluorescent images shown in Fig. 1B. qRT-PCR analysis of (B) *Nos2* and (C) *Arg1* expression in whole wounds of non-diabetic (grey bars) or diabetic (black bars) mice at the indicated time points following wounding. (D) Relative expression of Arg1:Nos2 in whole wounds from non-db (grey bars) and db (black bars) mice at time points indicated following wounding. (E) Analysis of general macrophage marker expression in db wounds, displayed as relative to non-db wounds, following reference gene normalization. For panels B-E, $n$=6, *$P$<0.05, **$P$<0.01, ***$P$<0.001.

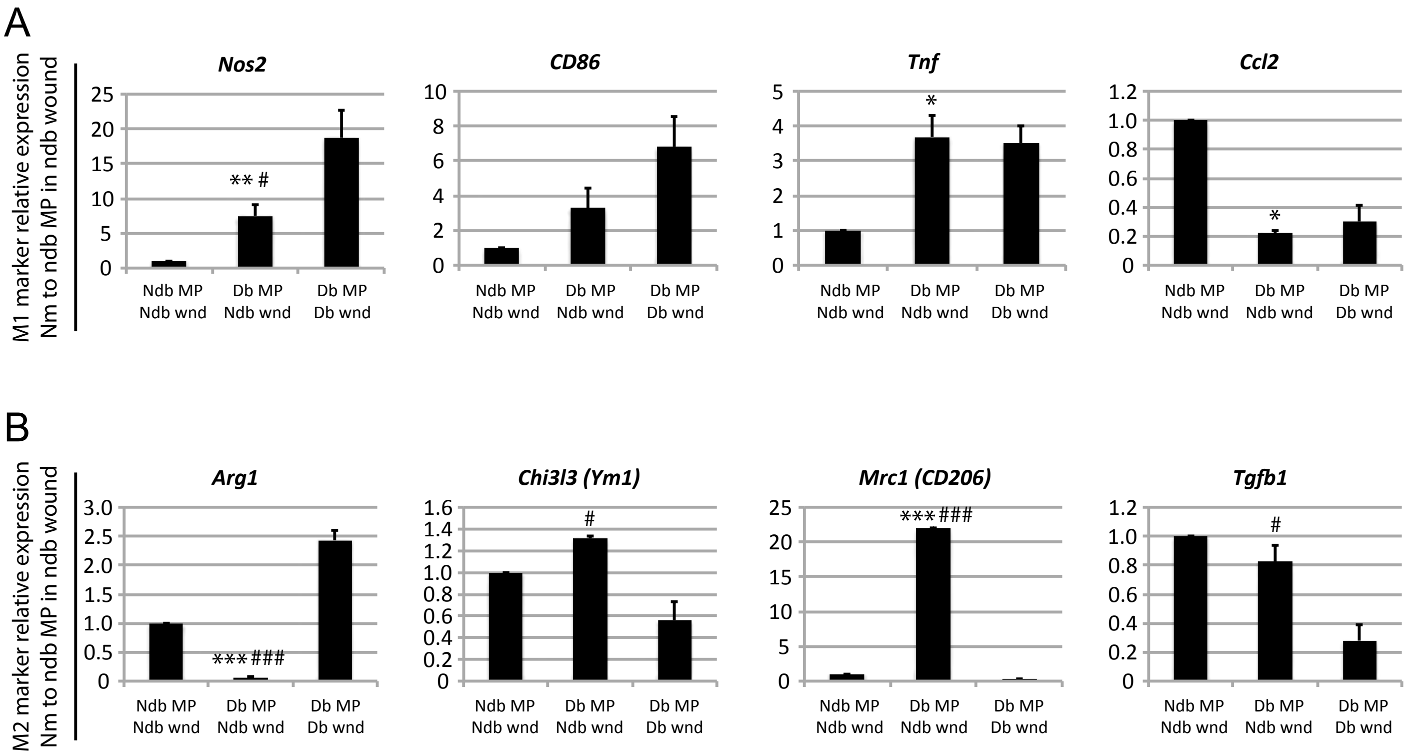


**Supplementary Fig. 2. Analysis of macrophage polarization markers following adoptive transfer of diabetic-derived macrophages into non-diabetic wounds.** (A) M1 marker gene analysis and (B) M2 marker gene analysis, comparing diabetic-derived macrophages (Db MP) in a non-db wound environment (Ndb wnd) with non-diabetic (Ndb) and diabetic (Db) controls injected into their respective environments. Assessment of significance between non-db and db in non-db wound environment is shown as *$P$<0.05, **$P$<0.01, ***$P$<0.001, assessment of significance between db in non-db and db wound environments is shown as #$P$<0.05, ##$P$<0.01, ###$P$<0.001.

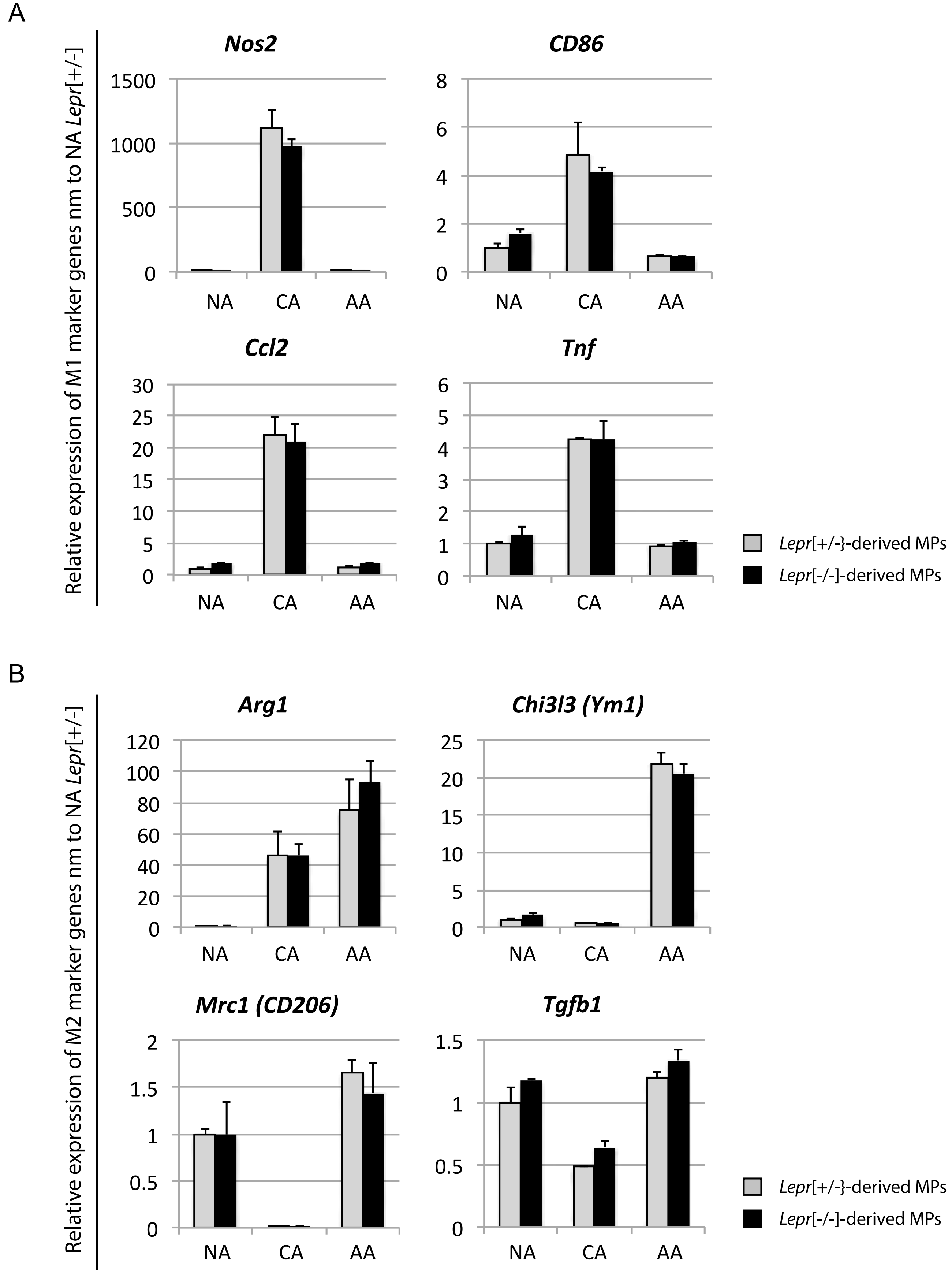


**Supplementary Fig. 3. Assessing the role of the leptin receptor in macrophage polarization.** qRT-PCR analysis of M1 (**A**) and M2 (**B**) marker gene expression in 3 week old *Lepr*$^{+/-}$ control pups and 3 week old *Lepr*$^{-/-}$ pups, prior to the onset of diabetes.

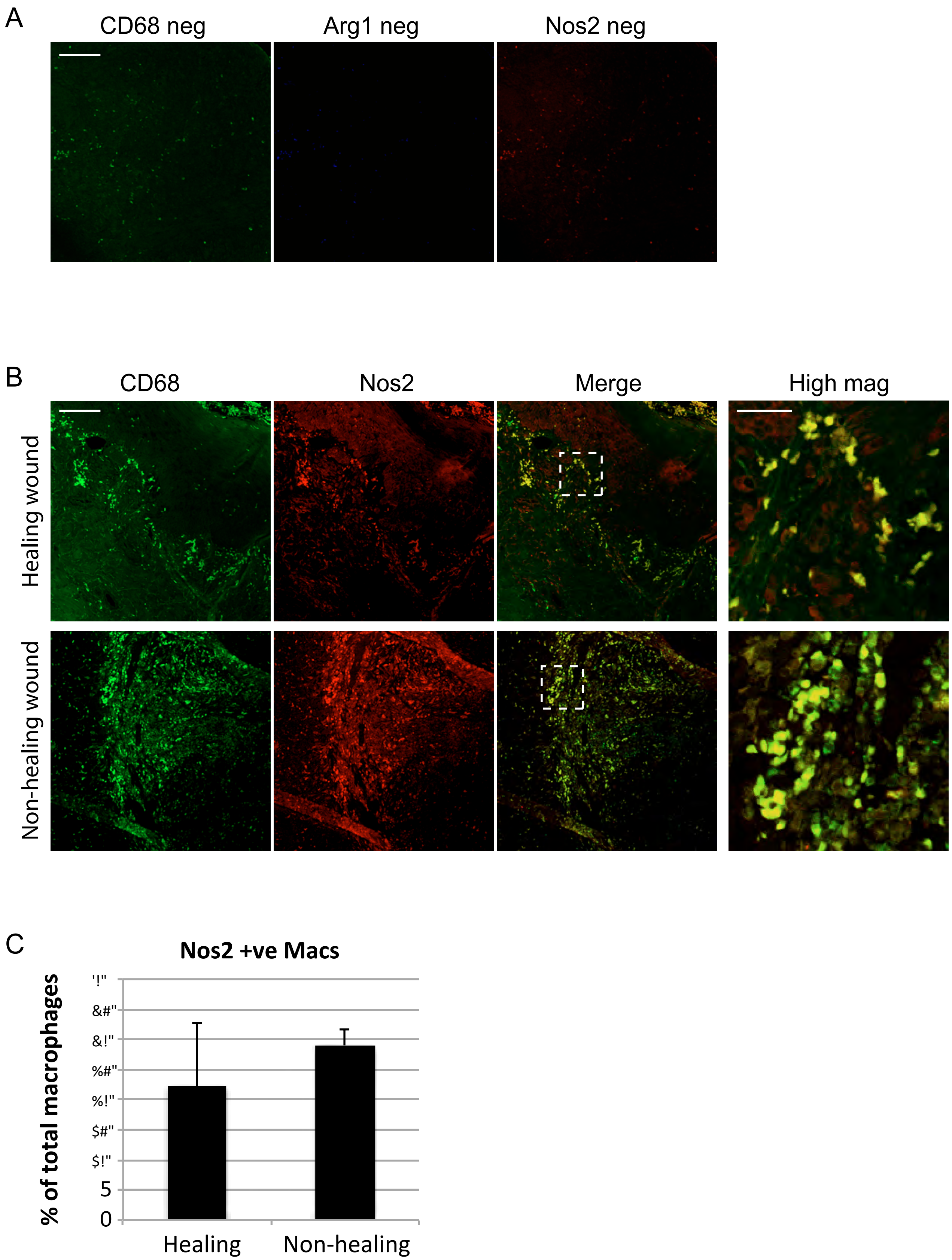


**Supplementary Fig. 4. Analysis of Nos2+ cells in human healing and non-healing diabetic foot ulcers.** (A) Negative controls (secondary only) for immunofluorescent images shown in Fig. 7 and this figure (scale bar=100 µm). (B) CD68 and Nos2 immunofluorescence in wound sections from healing and non-healing diabetic foot ulcers. The first three panels of each group show fluorescent staining as indicated (scale bar=100 µm). The fourth panel of each group shows a high magnification view of the area indicated by the white box in the merge panels (scale bar=25 µm). (C) Quantification of the percentage of macrophages that are $Nos2^{+}$ (double positive for Nos2 and CD68) in healing and non-healing wounds (*n*=7).

| Sample | Total # of single cell events | Total # of Gr-1$^+$/CD14$^-$ | Total # of Gr-1$^+$/CD14$^+$ | Total # of Gr-1$^-$/CD14$^+$ |
|---|---|---|---|---|
| D5 db A | 4092000 | 166000 | 667000 | 24000 |
| D5 db B | 2899000 | 86000 | 519000 | 22000 |
| D5 db C | 5128000 | 177000 | 682000 | 32000 |
| D5 non-db A | 3150000 | 99000 | 63000 | 15000 |
| D5 non-db B | 4118000 | 111000 | 210000 | 11000 |
| D5 non-db C | 2284000 | 53000 | 169000 | 1000 |
| D10 db A | 3937000 | 88000 | 374000 | 53000 |
| D10 db B | 4629000 | 100000 | 412000 | 24000 |
| D10 db C | 798000 | 17000 | 75000 | 1600 |
| D10 non-db A | 1400000 | 52000 | 14000 | 29000 |
| D10 non-db B | 2467000 | 32000 | 7400 | 21000 |
| D10 non-db C | 2222000 | 66000 | 20000 | 91000 |

**Supplementary Table 1.** Myeloid cell counts having the indicated immunophenotype from FACS data of day 5 (D5) and day 10 (D10) diabetic (db) and non-diabetic (non-db) wounds.
